# Insular Volume Reduction in Patients with Social Anxiety Disorder

**DOI:** 10.3389/fpsyt.2016.00003

**Published:** 2016-01-21

**Authors:** Akiko Kawaguchi, Kiyotaka Nemoto, Shutaro Nakaaki, Takatsune Kawaguchi, Hirohito Kan, Nobuyuki Arai, Nao Shiraishi, Nobuhiko Hashimoto, Tatsuo Akechi

**Affiliations:** ^1^Department of Psychiatry and Cognitive-Behavioral Medicine, Graduate School of Medical Sciences, Nagoya City University, Nagoya, Japan; ^2^Department of Psychiatry, Faculty of Medicine, University of Tsukuba, Ibaraki, Japan; ^3^Department of Psychiatry, Keio University, Tokyo, Japan; ^4^Department of Radiology, Toyota-Kai Medical Corporation, Kariya Toyota General Hospital, Kariya, Japan; ^5^Department of Radiology, Nagoya City University Hospital, Nagoya, Japan

**Keywords:** social anxiety disorder, magnetic resonance imaging, insula, voxel-based morphometry

## Abstract

Despite the fact that social anxiety disorder (SAD) is highly prevalent, there have been only a few structural imaging studies. Moreover, most of them reported about a volume reduction in amygdale, which plays a key role in the neural function of SAD. Insula is another region of interest. Its hyperactivity in regard to processing negative emotional information or interoceptive awareness has been detected in patients with SAD. Referring to these studies, we hypothesized that insular volumes might reduce in patients with SAD and made a comparison of insular volumes between 13 patients with SAD and 18 healthy controls with matched age and gender using voxel-based morphometry. As a result, we found a significant volume reduction in insula in the SAD group. Our results suggest that the patients with SAD might have an insular volume reduction apart from amygdala. Since insula plays a critical role in the pathology of SAD, more attention should be paid not only to functional study but also morphometrical study of insula.

## Introduction

Social anxiety disorder (SAD), also known as social phobia, is defined by excessive fear and avoidance of social situations. According to Clark and Wells (1), patients with SAD tend to have negative social cognitions in the situation in which they fear and these cognitions are enhanced by misperception of somatic symptoms.

Social anxiety disorder is one of the common psychiatric disorders (2) with a lifetime prevalence of 12% (3). Despite the fact that SAD is highly prevalent, there have been fewer structure MRI studies on SAD than mood disorders or other anxiety disorders. The first study was reported in 1994 by Potts et al. (4), which concluded that there was no statistically significant volume difference between 22 patients with SAD and controls by voxel-based morphometry (VBM) analyses. More than 15 years later, Irle et al. examined hippocampal/amygdala volumes of patients with SAD with region-of-interest (ROI) method and revealed hippocampal/amygdala volume reduction in patients with SAD (5). They also reported a negative correlation between a right hippocampal volume and Liebowitz Social Anxiety Scale (LSAS) and between a right amygdala volume and State-Trait Anxiety Inventory (STAI). Liao et al. conducted a study which integrated diffusion tensor imaging, resting-state functional magnetic resonance imaging (fMRI), and VBM (6). The study revealed a volume reduction in inferior temporal gyrus and parahippocampal/hippocampal gyrus and a volume increase in right medial prefrontal cortex (MPFC) in patients with SAD. Meng et al. also reported a volume reduction in bilateral thalami, right amygdala, and right precuneus, a negative correlation of a right amygdala volume with the duration of disease, and a positive correlation with the age of onset (7). They also suggested that the volume change can occur in the aberrant functional region. Although a few studies (8, 9) reported a volume increase in these regions, most of the studies mentioned a volume reduction in hippocampal/amygdala, which play key roles in the neural function of SAD according to the fMRI studies (10). Moreover, posttraumatic disorders (10, 11) and panic disorders (12–15), which are supposed to have a shared anxiety-related neural network with SAD, also present both hyperactivity and a volume reduction in amygdala. According to these perspectives, the same phenomenon could occur in patients with SAD. In other words, hyperactivity might accompany a volume reduction as Meg et al. mentioned.

In addition to hippocampus or amygdala, insula is another ROI. Insula is located in the central region of the cerebral, and it has connection with many cortical areas including anterior cingulate cortex (ACC), PFC, somatosensory areas, and limbic areas, such as amygdala (16). It has various roles, for instance, processing visceral sensory, visceral motor, vestibular, motor information (17), romantic love (18), and maternal love (19), and dealing with strong emotions, such as social pain (20) or a variety of negative emotions (21). Among those various roles, perceiving strong emotions and processing introspective awareness are major roles of insula (22). Previous fMRI studies in patients with SAD have detected hyperactivity of insula, and this has been related to the excessive processing of negative feeling, such as disgust or anger (10, 23–29). In those situations, insula is coactivated with amygdala and evaluates the emotional stimuli (30).

Another major role of insula is the interoception, which is defined as the awareness of the internal bodily state. When we feel heartbeat, or sense a touch or pain on skin, insula conveys these perceptions. This internal awareness is important to realize the bodily state or the image of the self (self-awareness) (31). Insula is involved in the excessive processing of the interoceptive state in patients with SAD, and this information is sent to the ACC, and ACC generates an altered prediction signal (32, 33). This signal causes the initiation of action, resulting in the safety behaviors or avoidance.

The network between insula and dorsal ACC has been known as “saliency network” (34, 35). This network is related to bottom-up detection of salient events, switching between other large-scale networks to facilitate access to attention and working memory resources when a salient event is detected, modulating autonomic reactivity to salient stimuli, and strong functional coupling with the ACC that facilitates rapid access to the motor system (34). According to the Clark and Wells model (1), which is the most well-known psychological model of SAD, patients with SAD tend to have negative social cognitions when they face the social situations and this leads to enhance the several somatic symptoms, such as palpitation, blush, and shivering. Then, they overestimate these symptoms as threatening matters and this cause much more negative social cognition (e.g., surrounding people might notice their somatic symptoms and people might think the patient is unworthiness). This can be viewed as inappropriate activation of saliency network. Indeed, there is a report which demonstrate decreased functional coupling of the insula within the saliency network in patients with SAD (36).

Considering this, insula could play such an important role in SAD. Therefore, we hypothesized that the insular volume is likely to reduce as well as the amygdala and hippocampus. A recent study reported a reduction of cortical thickness of insula in SAD by the ROI analyses (37), which is in line with our hypothesis. We investigated whether insular volume would reduce in patients with SAD in comparison with healthy individuals using VBM.

## Materials and Methods

### Subjects

All the patients were enrolled from the department of psychiatry at Nagoya City University Hospital. They met the criteria for generalized subtype SAD as the primary disorder, which were determined with the structured clinical interview for DSM-IV Axis I disorders (SCID-I). The control (CTL) group consisted of people who did not have concurrent and past Axis I disorders without the family history of psychiatric problems within the second degree relatives and were recruited from the local population. All the subjects were Japanese, right handed, which was confirmed based on the Edinburgh handedness inventory (38). We excluded the participants who had a history of major medical or neurological illness, significant head trauma, or a lifetime history of alcohol or drug dependence. As a result, the subjects recruited for this study consisted of 13 outpatients with SAD (five males and eight females; age range, 20–56 years; mean ± SD, 36.2 ± 11.8 years) and 18 healthy CTL individuals with similar age and sex (six males and 12 females; age range, 21–53 years; mean ± SD, 33.8 ± 9.6 years). The onset and duration of SAD (mean ± SD) were 13.0 ± 10.3 years old and 23.4 ± 14.4 years, respectively. Concurrent and past comorbidities of the patients were as follows: four patients had concurrent comorbid major depressive disorders, two had past major depressive disorders, one had a concurrent panic disorder, one had a past panic disorder, one had a concurrent bulimia nervosa, and one had a past bulimia nervosa and past adjustment disorder. Although there were some patients with concurrent Axis I disorders, SAD was their primary disorder, and those comorbid symptoms were under control to the extent that they managed to go through MRI scanning. More detailed characteristics of the participants are summarized in Table 1. The Ethics Committee of Nagoya City University Graduate School of Medical Sciences approved this study. After being explained about the study, all the subjects provided written informed consent.

**Table 1:** **Demographic data of subjects**.

		SAD group	CTL group	*p*
Number		13	18	
Gender	Male	5	6	1
	Female	8	12	
Age mean (SD)		36.2 (11.8)	33.8 (9.6)	0.5
IQ mean (SD)		106.2 (10.9)	109.3 (5.8)	0.3
LSAS mean (SD)		81.6 (14.3)	25.6 (14.2)	<0.001**
BFNE mean (SD)		35.3 (6.4)	18.6 (7.8)	<0.001**
SPS mean (SD)		37.2 (14.2)	7.1 (4.6)	<0.001**
SIAS mean (SD)		52.9 (10.1)	24.4 (10.2)	<0.001**
HRSD mean (SD)		8.8 (5.2)	0.8 (1.6)	<0.001**
Age of onset mean (SD)		13.0 (10.3)	NA	
Duration of SAD years mean (SD)		23.3 (14.4)	NA	
Antidepressants DDD mean (SD)		1.1 (0.8)	NA	
Antianxiety agents DDD mean (SD)		0.7 (0.8)	NA	
Hypnotics DDD mean (SD)		0.4 (0.9)	NA	
Antipsychotics DDD mean (SD)		0.1 (0.2)	NA	
Cognitive behavioral therapy (%)		9 (69.2)	NA	
Current comorbidity	Major depressive disorder (%)	4 (30.8)	NA	
	Panic disorder (%)	1 (7.7)	NA	
	Bulimia nervosa (%)	1 (7.7)	NA	
Past comorbidity	Major depressive disorder (%)	2 (15.4)	NA	
	Panic disorder (%)	1 (7.7)	NA	
	Bulimia nervosa (%)	1 (7.7)	NA	
	Adjustment disorder (%)	1 (7.7)	NA	
Gray matter volume mean (SD)		533.5 (50.9)	577.3 (72.6)	0.1
White matter volume mean (SD)		640.0 (52.3)	684.4 (88.1)	0.07

*SAD, social anxiety disorder; CTL, control group; IQ, Intelligence Quotient; LSAS, Liebowitz Social Anxiety Scale; BFNE, Brief Version of the Fear of Negative Evaluation Scale; SPS, Social Phobia Scale; SIAS, Social Interaction Anxiety Scale; HRSD, Hamilton Rating Scale for Depression; DDD, defined daily dose; NA, not applicable*.

**p<0.001.

### Psychiatric Assessment

The degrees of social anxiety were assessed by the LSAS, Social Interaction Anxiety Scale/Social Phobia Scale (SIAS/SPS), Brief Version of the Fear of Negative Evaluation Scale (BFNE), Hamilton Rating Scale for Depression (HRSD), and Japanese version of National Adult Reading Test (JART).

#### Liebowitz Social Anxiety Scale

Liebowitz Social Anxiety Scale is the most popular rater-administered assessment tool for the social anxiety with 24 performance or social interaction situations (39). It provides separate scores for fear (0–3 indicate none, mild, moderate, and severe, respectively) and avoidance (0–3 indicate never, occasionally, often, and usually, respectively) of the situation. High score indicates severe symptoms. Reliability and validity have been demonstrated sufficiently for both original and Japanese versions (40).

#### Social Interaction Anxiety Scale/Social Phobia Scale

Social Interaction Anxiety Scale/Social Phobia Scale are 20-item self-report questionnaires with ratings on a 4-point scale from 0 (not at all characteristic or true of me) to 4 (extremely characteristic or true of me) (41). Its total scores ranges from 0 to 80, and high score indicates severe symptoms. The SPS measures the fear of being observed, whereas the SIAS provides a measure of fear of social interaction. Sufficient reliability and validity have been demonstrated for both original and Japanese versions (42).

#### Brief Version of the Fear of Negative Evaluation Scale

Brief Version of the Fear of Negative Evaluation Scale (BFNE) assesses the fear of negative evaluation in social situations (43). The BFNE consists of 12 questions with a 1 (not at all characteristic of me) to 5 (extremely characteristic of me) point. Sufficient reliability and validity of the Japanese version have been reported in FNE (44).

#### Hamilton Rating Scale for Depression

Hamilton Rating Scale for Depression is the most frequently used clinician-administered ratings for depression (45). It contains 17 items and each item is scored on a 3- or 5-point scale, depending on the item. The total score estimates the severity of the depressive symptom. Reliability and validity have been demonstrated even in Japanese version (46).

#### Japanese Version of National Adult Reading Test

Japanese version of National Adult Reading Test was established according to the National Adult Reading Test (NART) (47). It contains 50 words reading *kanji* and estimates Intelligence Quotient (IQ). Reliability and validity have been demonstrated sufficiently.

All the rater assessments from psychiatric perspectives were carried out by a board-certified psychiatrist (Akiko Kawaguchi).

### MRI Image Acquisitions

Three-dimensional (3D) magnetization-prepared rapid gradient echo (MP-RAGE) images were acquired using a 3-T MR scanner (Magnetom Skyra, Siemens Medical Solutions, Erlangen, Germany) and a 32-channel head array coil. The parameters were as follows: repetition time, 7.3 ms; echo time (TE), 2.43 ms; flip angle, 9°; inversion time, 900 ms; matrix size, 256 × 256; slice thickness, 1 mm; and the number of sagittal images, 176 in total. A board-certified neuroradiologist (Takatsune Kawaguchi) reviewed all the scanned images and found no major abnormalities in any of the subjects.

### Demographic Data Analysis

Behavioral data was analyzed using SPSS version 19.0 software (IBM Corp. Armonk, NY, USA). Psychiatric measurements or demographic data were analyzed with unpaired *t*-tests for continuous variables and χ^2^ square test for categorical variables to compare the SAD and CTL groups. Results were considered statistically significant at *p* < 0.05.

### Imaging Data Analysis

All images were preprocessed and analyzed using Statistical Parametric Mapping 8 (SPM8^1^) running on Matlab R2013b (Mathworks Inv., Sherborn, MA, USA). VBM was performed using the VBM8 toolbox.^2^ Each MPRAGE image was segmented into gray matter (GM), white matter (WM), and cerebrospinal fluid (CSF). Subsequently, the segmented GM images were spatially normalized using the diffeomorphic anatomical registration through exponentiated lie algebra (DARTEL) algorithm. The voxel values were modulated by Jacobian determinants for the non-linear components (“non-linear only” option in VBM8 toolbox), and smoothed with an 8-mm full width at half maximum (FWHM) Gaussian kernel.

After preprocessing, two sample *t*-tests were performed between the SAD and the CTL groups to detect regional differences in GM images. We employed a small volume correction (SVC) within bilateral insulae based on our hypothesis. For the SVC analysis, a family-wise error (FWE)-corrected voxel level threshold of *p* < 0.05 was applied within bilateral insular ROIs to account for multiple comparisons of the results. ROIs of bilateral insulae were made using automated anatomical labeling (AAL) atlas included in WFU PickAtlas ver. 3.0.4 (48). Voxel coordinates were given in Montreal Neurological Institute (MNI) space and displayed as such.

## Results

### Demographic Data

Demographic and diagnostic characteristics of the participants were summarized in Table 1. Age, gender, and estimated IQ were not significantly different between the groups. On the other hand, SAD group showed significant higher scores than CTL in LSAS (SAD 81.6 ± 14.3; CTL 25.6 ± 14.2), SIAS/SPS (SAD 52.9 ± 10.1/37.2 ± 14.2; CTL 24.2 ± 10.2/7.1 ± 4.6), and BFNE (SAD 35.3 ± 6.4; CTL 18.6 ± 7.8).

### MRI Results

**Figure 1:**
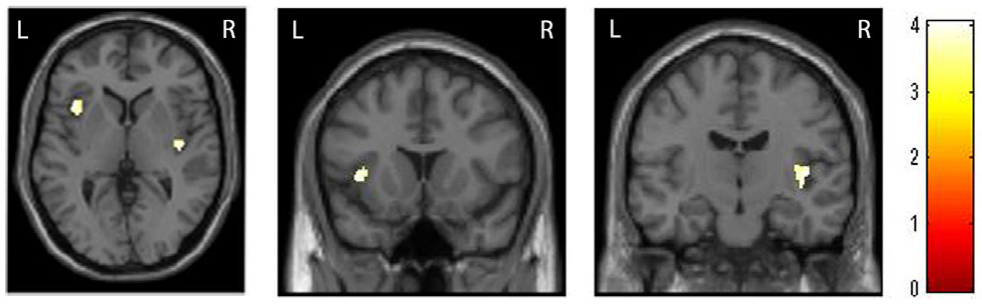
**Comparison of insular volumes between patients with social anxiety disorder (SAD) and the control (CTL) group**. Reduced bilateral insular volumes occurred in the SAD group as compared to the CTL group. For the statistical analysis, a family-wise error (FWE)-corrected voxel level threshold of *p* < 0.05 was applied within bilateral insular regions of interest to account for multiple comparisons of the results.

The whole-brain two sample *t*-tests showed no statistically significant differences between the SAD group and the CTL after multiple comparisons. Confining the ROIs to bilateral insulae, we found that left anterior and right posterior insular volumes significantly reduced in the SAD group as compared to the CTL (Figure 1; Table 2). We did not find significant correlation between insular volumes and LSAS, SIAS/SPS, or BFNE. We also investigated the difference in hippocampus and amygdala according to the previous reports, which resulted in no significant differences between the groups.

**Table 2:** **Comparison of insular volumes between patients with SAD and control**.

Structure	MNI coordinates			*T*	*p*	Cluster size
	*X*	*Y*	*Z*			
Right insula	42	−13	3	4.02	0.032	157
Left insula	−39	15	3	4.07	0.029	145

*MNI, Montreal Neurological Institute*.

## Discussion

### Main Findings

As we had expected, we found a statistically significant group difference in the insular volume between the SAD and the CTL groups. Bilateral insular volume was reduced in patients with SAD. There has been only one study investigating the relationship between insula and SAD, which reported cortical thinning of right insula (37). Our results reinforced it. Syal et al. implicated the altered interoception and aberrant insular-ACC connection in concern with interoception as reasons for the reduction.

Social anxiety disorder has been explained with cognitive behavioral model (1). According to the model, SAD patients tend to have negative social cognitions and these cognitions are enhanced by safety behaviors or somatic symptoms. Since this model focuses on negative cognition or feelings, most of the previous neuroimaging studies on SAD investigated mainly hippocampus or amygdala (10). Volume reduction or hyperactivation of these regions are also observed in other anxiety disorders, such as panic disorder (13–15).

In addition to the Clark and Wells model, misinteroception has been attracting attention. From the misinteroception point of view, SAD patients might recognize their somatic symptoms, such as blushing or trembling, as hazardous alarm to self, which reinforce their negative cognition. This aberrant interoception might be caused by the hyperactivity of insula because interoception is one of the major roles of the insula, and it links the bodily state and affective or cognitive processes (32). Indeed, anxiolytics are known to modulate those insular activations via GABA neuron and reduce the anxiety (49). Menon and Uddin pointed that hyperactivity of insula might be related to the enhanced detection of saliency (34).

Insula, especially anterior insula, has also known to be a center for saliency network. Saliency network comprises mainly of anterior insula and ACC. Anterior insula works as a hub, which integrates the external physiological sensation and sends signals to ACC in order to adjust the behavior (34).

Anterior insula evaluates the meaning of the external stimuli, and ACC generates the signals to PFC for controlling the behavior and cognition. Klumpp et al. detected the altered insula–ACC connection in SAD using fMRI connectivity analysis (26). They suggested that the less connectivity of insular-ACC in patients with SAD compared to the healthy comparisons and insufficient cognitive control of ACC and PFC affected the threat processing.

We observed volume reduction in not only anterior insula but also posterior insula. Posterior insular has reciprocal connections with higher order visual areas, auditory processing areas, somatosensory areas as well as anterior insula, and its function is specialized for directly experienced, multimodal sensory processing, particularly somatosensory processing (16). Nagai et al. also points that dysfunction of the posterior insular cortex might lead to visual–somatosensory imbalance, resulting in depersonalization or agoraphobia (17). Taken together, hyperactivity and dysfunction of saliency network and posterior insula might contribute the pathophysiology of SAD as well as hippocampus/amygdala regions.

As mentioned above, hyperactivity of insula is crucial in the neural pathology of SAD, and Meng et al. suggested that a volume change could occur in the aberrant functional region (7). Our results were in line with this idea. There are some functional and structure studies, which show both hyperactivity and a volume reduction in SAD as well as in other anxiety disorders (e.g., similar insular hyperactivity and volume reduction have found in panic disorder, which was also applied the cognitive behavioral model) (13–15). Furthermore, we suggest that in future studies, the effect of pharmacological or psychological treatment, or characteristics of patients on an insular volume should be investigated in large samples. Since treatment reduces the hyperactivity of insula, we expect that accompanied volume reduction might be recovered.

Though previous studies report volume reduction in hippocampus or amygdala, we did not find significant volume reduction in our sample. One of the reasons might be due to the small sample size. Indeed, if we lower the statistical threshold, group comparisons show volume reduction in the amygdala/hippocampal regions of SAD patients compared to controls (*p* < 0.005, uncorrected for hippocampus and *p* < 0.05 uncorrected for amygdala). Taking this into consideration, we suggest that both amygdala/hippocampus and insula contribute to the underlying mechanism of SAD.

In this study, we employed VBM to investigate the volume reduction in SAD. A previous study reported a reduction of cortical thickness of insula in SAD (37). Hutton et al. compared cortical thickness and VBM and suggested that these should be considered as complementary approaches because VBM is additionally sensitive to local surface area and cortical folding as well as cortical thickness (50). Considering this, our results using VBM reinforce the previous report in that insula is involved in the pathophysiology of SAD.

### Limitations

The present study had several limitations. First, some of the participants with SAD had comorbid mood, anxiety, and eating disorders. However, their primary diagnosis was SAD and, as summarized in Table 1, their HRSD scores were in a low level. Second, the effect of the antidepressant and benzodiazepine medications of the patients might have interfered with the outcome. However, there is a large study which shows that the medication does not have much effect on volumetric results among the patients with same severity (51). Furthermore, our study was based on a small sample size, and this might have been the reason for us not to have been able to find a significant group difference in the whole brain analyses. Our result needs to be replicated in a future study with a larger sample.

Although there are several limitations, we suggest the possibility that insula, which plays a critical role in the pathology of SAD, has a volume reduction as well as in amygdala. Considering the importance of the functional role of insula, we should pay more attention to morphometrical study of insula.

## Author Contributions

AK designed and managed the study. Image analyses were carried out by AK and TK. KN and SN supervised the image analyses. TK performed the quality control of MRI. HK and NA contributed to MRI data acquisition as radiology technicians. NS and NH managed the patient enrollment. AK carried out statistical analyses and drafted the manuscript under the supervision of KN, SN, and TA. All authors read and approved the final manuscript.

## Conflict of Interest Statement

The authors declare that the research was conducted in the absence of any commercial or financial relationships that could be construed as a potential conflict of interest.

## Footnotes

^1^http://www.fil.ion.ucl.ac.uk/spm/

^2^http://dbm.neuro.uni-jena.de/vbm
